# Septin 9 has Two Polybasic Domains Critical to Septin Filament Assembly and Golgi Integrity

**DOI:** 10.1016/j.isci.2019.02.015

**Published:** 2019-02-19

**Authors:** Mohyeddine Omrane, Amanda Souza Camara, Cyntia Taveneau, Nassima Benzoubir, Thibault Tubiana, Jinchao Yu, Raphaël Guérois, Didier Samuel, Bruno Goud, Christian Poüs, Stéphane Bressanelli, Richard Charles Garratt, Abdou Rachid Thiam, Ama Gassama-Diagne

**Affiliations:** 1 INSERM, Unité 1193, Villejuif 94800, France; 2 Université Paris-Sud, UMR-S 1193, Villejuif 94800, France; 3 Laboratoire de Physique de l’Ecole Normale Supérieure, ENS, Université PSL, CNRS, Sorbonne Université, Université Paris-Diderot, Sorbonne Paris Cité, Paris, France; 4 Instituto de Física de São Carlos, Universidade de São Paulo, São Carlos, Brazil; 5 Institute for Integrative Biology of the Cell (I2BC), CEA, CNRS, Université Paris-Sud, Université Paris-Saclay, 91198 Gif Sur Yvette Cedex, France; 6 Commissariat à l’Energie Atomique et aux Energies Alternatives, 91191 Gif-Sur- Yvette Cedex, France; 7 Institute Curie, PSL Research University, CNRS UMR 144, Paris, France

**Keywords:** Membrane Architecture, Molecular Interaction, Cell Biology, Functional Aspects of Cell Biology

## Abstract

Septins are GTP-binding proteins involved in several membrane remodeling mechanisms. They associate with membranes, presumably using a polybasic domain (PB1) that interacts with phosphoinositides (PIs). Membrane-bound septins assemble into microscopic structures that regulate membrane shape. How septins interact with PIs and then assemble and shape membranes is poorly understood. Here, we found that septin 9 has a second polybasic domain (PB2) conserved in the human septin family. Similar to PB1, PB2 binds specifically to PIs, and both domains are critical for septin filament formation. However, septin 9 membrane association is not dependent on these PB domains, but on putative PB-adjacent amphipathic helices. The presence of PB domains guarantees protein enrichment in PI-contained membranes, which is critical for PI-enriched organelles. In particular, we found that septin 9 PB domains control the assembly and functionality of the Golgi apparatus. Our findings offer further insight into the role of septins in organelle morphology.

## Highlights

• Two polybasic domains mediate septin 9 interactions with PIs
• Human septins have amphipathic helices suitable for membrane binding
• Septin 9 polybasic domains mediate the formation of septin higher-order structures
• The mutation or depletion of septin polybasic domains induces Golgi fragmentation

## Graphical Abstract

**Figure:**
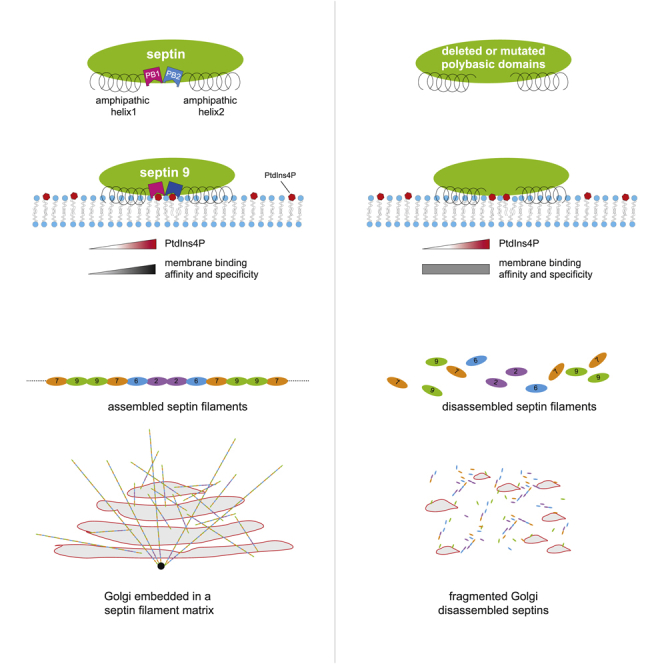

## Introduction

Septins form a GTPase protein family that is found in eukaryotes from yeasts to animals but is absent in higher plants and certain protists (Pan et al., 2007, Nishihama et al., 2011). In mammals, 13 septins have been identified and placed in four classes (the septin 2, 3, 6, and 7 subgroups) (Peterson and Petty, 2010). Septins assemble into apolar complexes that are able to form higher-order structures such as filaments and rings (Weirich et al., 2008). Each septin has at least two interfaces: one interface contains GTP-binding domain motifs, referred to as the G interface, and the other contains the N and C termini of the protein, called the NC interface. Thus two septin proteins can develop G/G or NC/NC interactions with neighboring septins. Septins thereby form hetero-oligomeric complexes made up of hexameric subunits with the following sequence: (G7NC/NC6G/G2NC/NC2G/G6NC/NC7G) (Sirajuddin et al., 2007). Septin 9 assembles at the extremities of the hexamer to generate an octamer (Kim et al., 2011). This octamer has the NC interface of septin 9 at its ends, i.e., NC9G/G7NC/NC6G/G2NC/NC2G/G6NC/NC7G/G9NC, and is the building block for higher-order septin structures (Sirajuddin et al., 2007, Sellin et al., 2011, Kim et al., 2011). During membrane remodeling processes, these structures can act as a diffusion barrier or scaffold that recruits cytosolic proteins and other cytoskeletal elements such as microtubules or actins (Tanaka-Takiguchi et al., 2009, Fung et al., 2014, Bridges et al., 2016, Mostowy and Cossart, 2012).

Septins bind specifically to phosphoinositides (PIs) via a polybasic domain (PB1) located at the N terminus of their GTP-binding domain. This interaction with PIs supposedly mediates septin membrane association, which is a determining factor for the structural and functional features of the protein (Pan et al., 2007, Zhang et al., 1999, Tanaka-Takiguchi et al., 2009, Casamayor and Snyder, 2003). Septins associate with a variety of PIs at different intracellular membranes (Zhang et al., 1999, Akil et al., 2016, Dolat and Spiliotis, 2016, Pagliuso et al., 2016) and control numerous cellular functions such as cytokinesis, ciliogenesis, vesicular trafficking, cell polarity, and lipid droplet formation (Fung et al., 2014, Oh and Bi, 2011, Song et al., 2016, Balla, 2013, Gassama-Diagne and Payrastre, 2009, Gassama-Diagne et al., 2006, Akil et al., 2016, Schink et al., 2016).

The morphology and positioning of intracellular organelles such as the Golgi and ER are critical for the proper transport and delivery of vesicles to maintain cell polarity, tissue homeostasis, and functions (Lavieu et al., 2014, de Forges et al., 2012, van Bergeijk et al., 2016). Such morphological arrangements are often ensured by cytoskeletal factors such as microtubules and actins, which are in part recruited by septins (Tanaka-Takiguchi et al., 2009, Fung et al., 2014, Bridges et al., 2016, Mostowy and Cossart, 2012). However, whether septins directly affect organelle morphology and function is poorly understood (Gurel et al., 2014, Weirich et al., 2008).

Here, by using septin 9 crystal structures and simulations of the molecular dynamics (MD) of the septin 9 monomer and dimer, we have identified the existence of a second polybasic domain (PB2). The deletion of PB2 phenocopied PB1 deletion by reducing the binding capacity of septin 9 to PI lipids and impairing the formation of septin filamented structures, hereinafter referred to simply as filaments. However, *in vitro* flotation assays revealed that the PB domains are not required for septin 9 membrane binding but proffer specificity to PI-containing membranes. These findings prompted us to identify amphipathic helices that are adjacent to the PB domains and possibly mediate the physical association of septin 9 with membranes. We studied the importance of the PB domains on organelles and determined their critical role in Golgi assembly and functionality.

## Results

### Septins Have a Second Polybasic Domain PB2 that Forms with PB1 a Basic Cluster at the NC Interface

**Figure 1:**
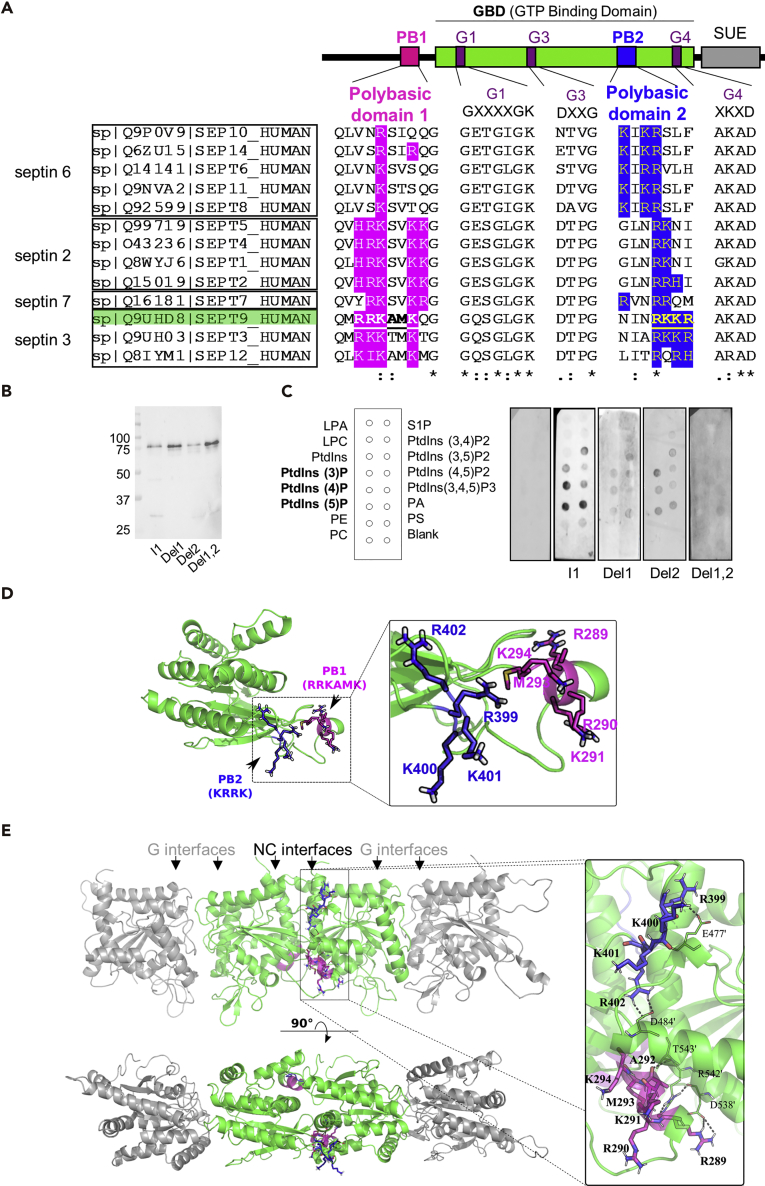
Septin 9 and Human Septins Have Two Polybasic Domains (A) Top: Schematic representation of the organization of septin domains. Bottom: Multiple alignments of human septins: the polybasic domain 1 (PB1) and polybasic domain 2 (PB2) are highlighted in magenta and blue, respectively. Human septin subgroups are shown in boxes. (B) Western blot of purified septin 9_i1, septin 9_del1, septin 9_del2, and septin 9_del1,2. (C) PIP strip overlay assay: PIP strips were incubated with either purified septin 9_i1 (I1), septin 9_del1 (Del1), septin 9_del2 (Del2), or septin 9_del1,2 (Del1,2) proteins at 0.5 μg/mL or with the V5 tag peptide as a negative control and analyzed using the anti-V5 antibody. LPA, lysophosphatidic acid; LPC, lysophosphocholine; PtdIns, phosphatidylinositol; PtdIns(3)P; PtdIns(4)P; PtdIns(5)P; PtdIns(3,4)P2; PtdIns(3,5)P2; PtdIns(4,5)P2; PtdIns(3,4,5)P3; PA, phosphatidic acid; PS, phosphatidylserine; PE, phosphatidylethanolamine; PC, phosphatidylcholine; S1P, sphingosine 1-phosphate. (D) Model of septin 9 based on the crystal structure (PDB code 5cyp) showing PB1 and PB2. (E) Model of the septin G9NC/NC9G complex using the simulated dimer of septin 9 at the NC interface and based on the symmetry operations of the crystallographic structure (PDB code 5cyp). The two molecules of septin 9 on either side of the NC interface are in green, and their encompassed PB1 and PB2 are presented in magenta and blue, respectively. The rectangle indicates PB1 and PB2 shown at a higher magnification on the right. The residues for PB1 and PB2 are labeled and outlined in black. Dashed black lines indicate the interaction between the PBs and neighboring septin 9 residues.

Septins bind to PI lipids via a polybasic domain (PB1) located at the N terminus of their GTP-binding domain (Zhang et al., 1999, Pan et al., 2007). However, we recently found that the deletion of PB1 in septin 9 reduces, but does not abolish, the interaction between septin 9 and monophosphorylated PIs (Akil et al., 2016). This observation prompted us to look for the presence of additional PI-interacting domains. We aligned the sequences of septin 9 and other human septins and identified a second motif enriched in basic amino acids (aa 399–402 of human septin 9 isoform 1; 586 residues) (Figure 1A). This second polybasic domain, which we termed PB2, contains a variable number of basic amino acids (2–4), but is conserved in all human septins (Figure 1A).

We next generated and purified a PB2-deleted mutant (septin 9_del2), a PB1-deleted mutant (septin 9_del1) (Akil et al., 2016), and a mutant lacking both PB1 and PB2 (septin 9_del1,2) (Figure S1A). These proteins displayed band profiles similar to septin 9_i1 (Figures 1B and S1B), which was in a monomeric form based on migration on a native gel (Figure S1C). We then used a phosphatidylinositol phosphate (PIP) strip overlay assay to determine the affinity of septin 9_i1 and its mutant forms with different phospholipid head groups. As expected, we found a specific interaction between septin 9_i1 and phosphatidylinositol (PtdIns) monophosphate (Figure 1C). The interaction signal with PIs was decreased in septin 9_del1 and septin_del2 and was almost abolished in septin 9_del1,2 (Figure 1C). This result supports the idea that both PB domains can mediate the interaction of septin 9 with PIs.

To study the involvement of PB2 in the structural organization of septin 9, we opted for an MD simulation approach using the most resolved septin 9 crystal structure (aa 293–564), PDB code 5cyp. In this structure, the missing residues and side chains were added and completed by amino acids from 276 to 292 (see Methods), which included those of PB1. Regardless of the initially folded state of these added residues, we found one single final equilibrium conformation of the protein where the N-terminal region was pre-folded into an α-helix around PB1 (Figures 1D and S1D). This equilibrated monomer was then superimposed on the crystal structure of septin 9 (PDB: 5cyp) to build a tetramer that contains the NC-NC interface (Figure 1E). At this interface, PB2 and PB1 appeared to make numerous salt bridges between septin monomers (Figure 1E, black dots in the inset); we found optimal distances between amino acids of the PB domains and those of the adjacent septin. These interactions involved the R399 and R402 residues of PB2 interacting, respectively, with E477′ and D484′, and the R289 residue of PB1 interacting with D538′. The main chain atoms of R289 and A292 in PB1 made hydrogen bonds with R542′ and T543′ of the neighboring septin (Figure 1E). This structural analysis indicates that the PB2 domain forms an extended basic cluster with PB1 at the NC interface of septin 9.

### Contribution of PB Domains to Septin 9 Membrane Association

To determine whether the PB domains are equally involved in septin 9 membrane interactions, we first performed MD simulations of the interaction between the monomeric structure of the protein shown in Figure 1D and membranes devoid of PtdIns(4)P (Figure S2A). The simulations were made by leaving the protein close to a dioleoylphosphatidylcholine (DOPC) membrane, thus offering it the opportunity to change conformation over time. We simulated three conditions by changing the initial protein conformation and velocity (Figure S2A, MD1-3). In all cases, septin 9 was recruited to the membrane. However, we found that PB1 was always in contact with the membrane (Figure S2A) (see Transparent Methods), whereas PB2 was not under one of the conditions (Figure S2A, MD1). This observation suggests that PB1 may be better positioned to interact with membranes.

**Figure 2:**
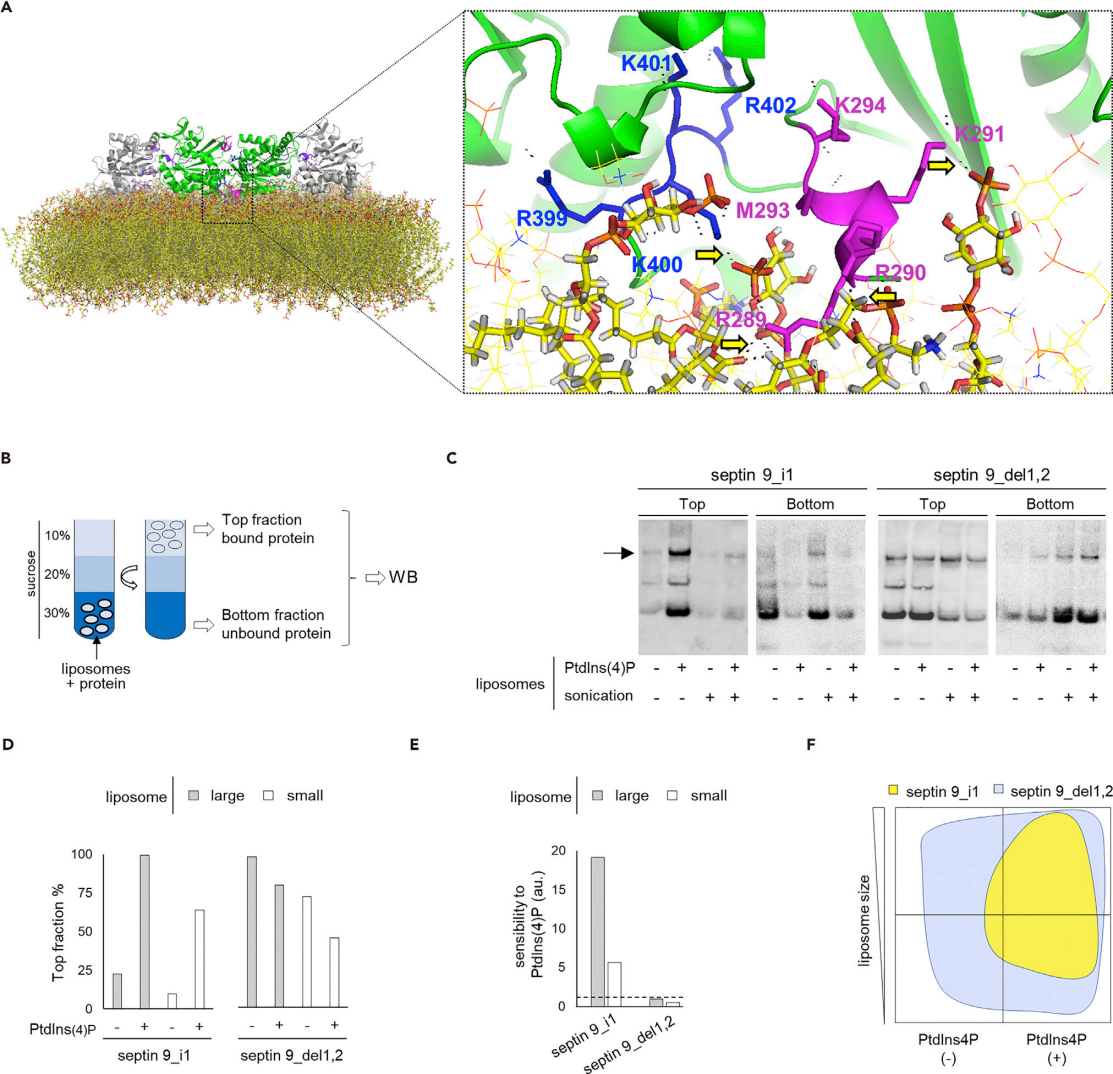
PBs Are Required for Specific Interactions between Septin 9_i1 and PIs and the Recognition of Membrane Forms (A) Modeling of the septin 9 complex interaction with a patch of PI-containing lipid bilayer. The dotted rectangle indicates the PB1-PB2 area proximal to the membrane shown in higher magnification. The residues for PB1 and PB2 are labeled. Yellow arrows indicate the interaction between PBs and the membrane. (B) Schematic representation illustrating the liposome floatation assay. (C) Western blots of septin 9_i1 (I1) and septin 9_del1.2 (Del1,2) subjected to a liposome flotation assay; the arrow indicates the band corresponding to V5-tagged Septin9_i1 (68 kDa), which was further analyzed. (D) Bar graph representing the percentage of protein in the top fraction (bound protein) from an analysis of the blots presented in (B). (E) Bar graph representing the sensitivity of the protein to PtdIns(4)P (the ratio of protein bound with PtdIns(4)P(+) liposomes to protein bound with PtdIns(4)P(−) liposomes). Dashed line indicates the 1 value. (F) Schematic phase diagram illustrating the results of western blot analysis of the liposome flotation assay.

To further clarify the involvement of PB1 and PB2 in septin 9 interactions with PI lipids, we took advantage of the recently resolved crystal structure of septin 3 (Macedo et al., 2013), which belongs to the same subgroup as septin 9 (Figure 1A). By using homology modeling, we built a septin 9 monomer and tetramer (Figures S2B and S2C), and used the tetramer to determine its spatial organization on a DOPC/DOPE bilayer containing PtdIns3P, PtdIns4P and PtdIns(5)P (Lomize et al., 2012). In the membrane-proximal PB1-PB2 cluster of the tetramer, we found that the basic residues R^289^, R^290^, K^291^, and K^400^ were particularly well positioned to interact with the phosphate head group of the PIs (Figure 2A). Three of these residues, namely, R289, R290, and K291, belong to PB1, and K400 to PB2. This analysis also supports the idea that PB1 is more closely involved in regulating septin 9 membrane binding than PB2, which was consistent with the previous MD results (Figure S2A).

### Septin 9 Interactions with Membranes *In Vitro* Do Not Require Any of the PB Domains

We decided to study the contribution of PB domains to septin association *in vitro*. Large and small liposomes (e.g., above 300 nm and below 200 nm), containing PtdIns(4)P or not, were, respectively, generated by vortex and high-power sonication, so as to mimic flat and curved membranes. The liposomes were then mixed with septin 9_i1 and septin 9_del1,2. A sucrose gradient flotation assay was subsequently performed (Figure 2B), during which only bound proteins would float up with the liposomes to the top fraction (Figure 2B). The supernatant fraction was collected and the amount of bound proteins determined by western blot (Figure 2C).

As expected, septin_i1 bound more strongly to liposomes containing PtdIns(4)P than to those that lacked PtdIns4P (Figures 2C–2F). However, binding was reduced on smaller liposomes (Figures 2C–2F and S2D), which suggests that the binding capacity of septin 9 was better with flat rather than positively curved membrane regions. Surprisingly, we found that septin 9_del1,2, which lacked both PB domains, bound efficiently to all membranes (Figures 2C–2F), despite the fact that it also had a slight preference for flatter membranes (Figure S2D). This efficient binding of septin 9_del1,2 was not detected in the PIP strip overlay assay because perfect membrane bilayers are not generated using this method (Figure 1C); it was, however, consistent with the binding of septin 9 to membranes devoid of PI lipids obtained by MD simulations (Figure S2A). Finally, the binding of septin 9_del1 and septin 9_del2 was not enhanced by the presence of PtdIns(4)P, and it was almost lost on small liposomes (Figure S2E). These results suggest that PB1 and PB2 are both required for the specific and efficient binding of septin 9 to PI-containing membranes.

In conclusion, our data described above, and particularly those obtained with septin 9_del1,2, suggest that septin 9 can bind bilayer membranes without involving its PB domains. Both PB domains seem to be necessary to provide septin 9 binding selectivity to PI-containing membranes, and especially to flat membranes or at least to those that are not positively curved.

### Septins Have PB-Adjacent Amphipathic *α*-Helix

**Figure 3:**
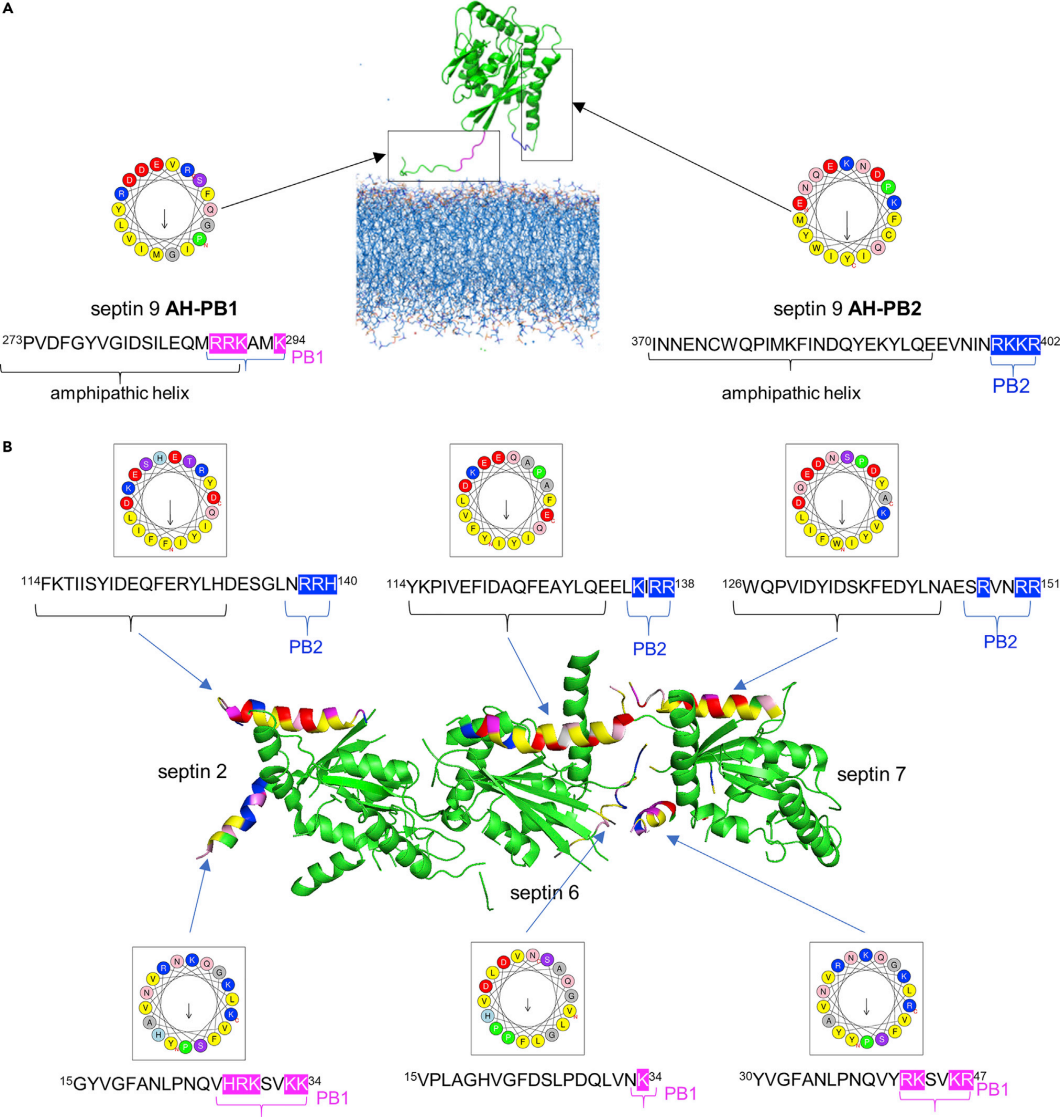
Septin 9 Has Putative PB-Associated Amphipathic Helices Mediating Its Binding to the Membrane (A) Crystal structural model of septin 9 (PDB code 5cyp) showing the predicted amphipathic helices opposed to PB1 and PB2 and their helical wheel representation generated using Heliquest. (B) Structural model of the (NC2G/G6NC/NC7G) septin complex published by Sirajuddin et al. showing the predicted amphipathic helices opposed to PB2 and the helical wheel representation of these helices generated using Heliquest. The sequence of each predicted helix is presented; the corresponding helical wheel and PB1 basic residues are highlighted in magenta and those of PB2 in blue.

We next tried to determine how septin 9 was able to interact directly with membranes in the absence of PI lipids and without its PB domains (Figure 2). Many soluble proteins bind membranes using amphipathic alpha-helix (AH) motifs, which are, moreover, able to detect subtle differences in membrane curvature and charges (Bigay and Antonny, 2012), as is the case for septin 9. We therefore performed a bioinformatics screening for AHs in the full sequence of septin 9. Two striking sequences emerged from our analysis as being the most prominent AHs (aa 274 to 294 and aa 370 to 402). Surprisingly, these AHs were directly adjacent to PB1 and PB2, respectively (Figure 3A). The sequence close to PB1 corresponded to a flexible strand that can fold into an AH, which is probably why it is missing from the septin 9 crystal structure. MD simulation showed that this portion of the protein indeed folded as an α-helix (Figure S1D) and was well positioned to bind membranes (Figure 3A). The AH close to PB2 was folded but oriented toward the interior of the protein, unless a conformational switch occurred. These AHs contain very hydrophobic residues, such as tyrosine, tryptophan, and phenylalanine, a feature that enhances membrane association. Our analysis therefore suggested that septin 9 has at least one PB-adjacent AH associated with PB1 that can mediate its physical association with membranes. In other septins, we also found flagrant AHs juxtaposed with the PB2 of septins 2, 6, and 7 with which septin 9 interacts to form the octamer. We took advantage of the existence of a crystal structure of the trimer formed by septins 2, 6, and 7 to verify the orientation of the AHs. We found that the AH-PB2 of septin 6 was suitably oriented to bind membranes (Figure 3B). The AH feature at the PB1 domain of these septin 9 counterparts was less pronounced (Figures 3B and S3A–S3D).

### Septin 9 PB Domains Are Essential for the Formation of Septin Filaments

Our data supported the idea that the AHs of septin 9 are probably the membrane binding motifs that are restricted to PI-containing membranes by the PB domains. Under this scenario, the PB domains would be more available to participate in the NC/NC interactions that mediate the formation of septin higher-order structures.

**Figure 4:**
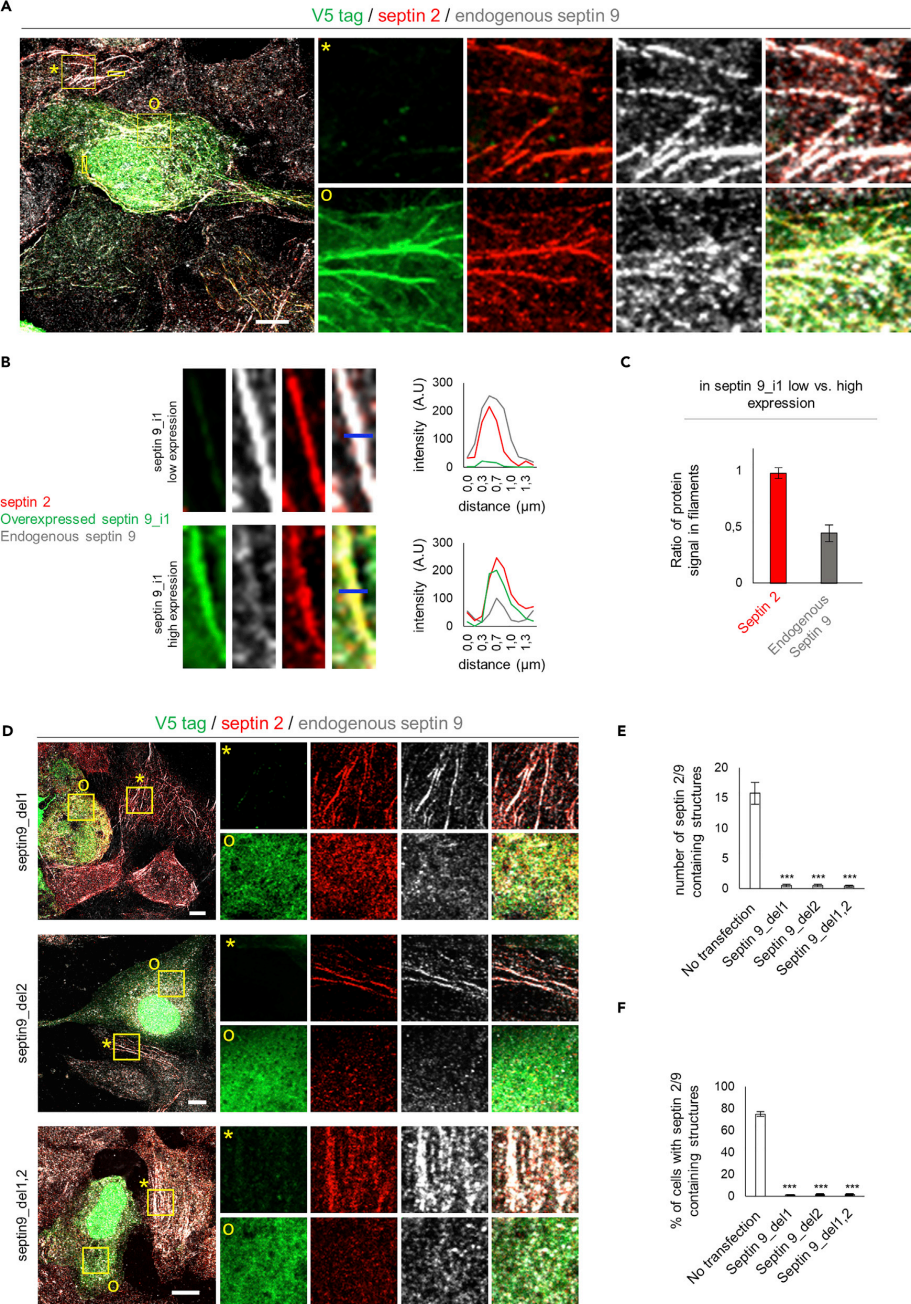
Overexpressed Septin 9_i1 Replaces Indigenous Septin 9 in Septin Filaments, Whereas PB-Mutated Septin 9 Expression Impairs Them (A) Huh7,5 cells transfected with septin9_i1 for 48 h and then fixed and stained for V5 tag in green (septin 9_i1), endogenous septin 2 (red) and endogenous septin 9 (gray). * indicates low septin9_i1-expressing cells and O indicates high septin9_i1-expressing cells. Squares indicate the area shown at higher magnification on the right. Scale bar, 10 μm. (B) Higher-magnification images of the small yellow rectangles shown in (A). On the right, the line graphs show the line profile analysis of the lines shown in these images. (C) Bar graph showing the ratio of the intensity of endogenous septin 2 and endogenous septin 9 in low septin 9_i1-expressing cells to those of high-septin 9_i1-expressing cells. Values are mean ± SEM from 10 filaments under each condition from two independent experiments. (D) Huh7,5 cells transfected with septin 9_del1 (Del1), septin 9_del2 (Del2), or septin 9_del1,2 (Del1,2) for 48 h and then fixed and stained for V5 tag (green), endogenous septin 2 (red), and endogenous septin 9 (gray). * indicates a low-expressing or non-transfected cell, and O indicates a transfected cell. Squares indicate the area shown at higher magnification to the right. (E) Bar graph representing the number of the filament structures containing both endogenous septin 9 and septin 2. Values are mean ± SEM from 10 cells under each condition from two independent experiments *** P<0.0001, (Student’s t-test). (F) Bar graph representing the percentage of cells containing filament structures of endogenous septin 9 and septin 2. Values are mean ± SEM from 50 cells under each condition from two independent experiments *** in indicates P<0.0001, (Student’s t-test).

We transfected HeLa cells with septin 9_i1, which we found was incorporated in higher-order filamentous structures that also contained endogenous septin 9 and septin 2 (Figures 4A and 4B). However, the strong expression of septin 9_i1 seemed to displace endogenous septin 9 from the filaments, whereas septin 2 remained recruited at a similar level (Figures 4B and 4C). Our overexpressed septin 9 construct thus had a dominant negative effect on endogenous septin 9 (Figure S4E). When cells were transfected with the PB-deleted constructs (Del1, Del2, Del1,2), the filamentous structures were lost (Figures 4D–4F) despite the presence of endogenous septin 9. Here also, these constructs displayed the dominant-negative effect of septin 9 (Figure S4E), and septin 2 was not present in the filaments (Figure 4D). These results suggest that both PB domains are involved in septin 9 assembly, in line with our previous results from the structural analysis (Figures 1E and S2C).

To avoid possible conformational changes to the protein and its dysfunction because of the deletion of PB domains, we performed simple point mutations. We substituted the basic lysine and arginine amino acids (K and R) with glutamine (Q), which is a non-charged but polar amino acid that has a long side chain as in K and R; this substitution was therefore optimal to minimize possible conformational changes in the protein. For example, in septin 9_Q1, the R and K residues of PB1 were not deleted as in septin 9_del1, but replaced by Q. We observed that cells transfected with septin 9_Q1, septin 9_Q2, or septin 9_Q1,2 were unable to form septin filaments (Figures S4A–S4C), in the same way as with the deletion (Figures 4D–4F). We next performed simple mutations by substituting one or two R with alanine (A) within PB1, and the filaments were lost once again (Figure S4D). None of these constructs affected the normal expression of endogenous septin 9 (Figure S4E).

In conclusion, the point mutant phenocopied the deletion constructs, and the results suggest that the positively charged PB cluster at the NC/NC interface is essential to the formation of septin filaments.

### Septin 9 PB Domains Are Critical for Golgi Assembly

Septin 9 binds mainly to PtdIns(3)P, PtdIns(4)P, and PtdIns(5)P, which are primarily detected in early endosomes (EE), the Golgi apparatus, and the ER, respectively (Pendaries et al., 2006, Kutateladze, 2010, Sarkes and Rameh, 2010). We thus wanted to probe whether PB1 and PB2 contribute to the organization of these endomembrane compartments. We expressed septin 9_i1 and the mutant constructs in HeLa cells and then studied the morphology of these organelles.

**Figure 5:**
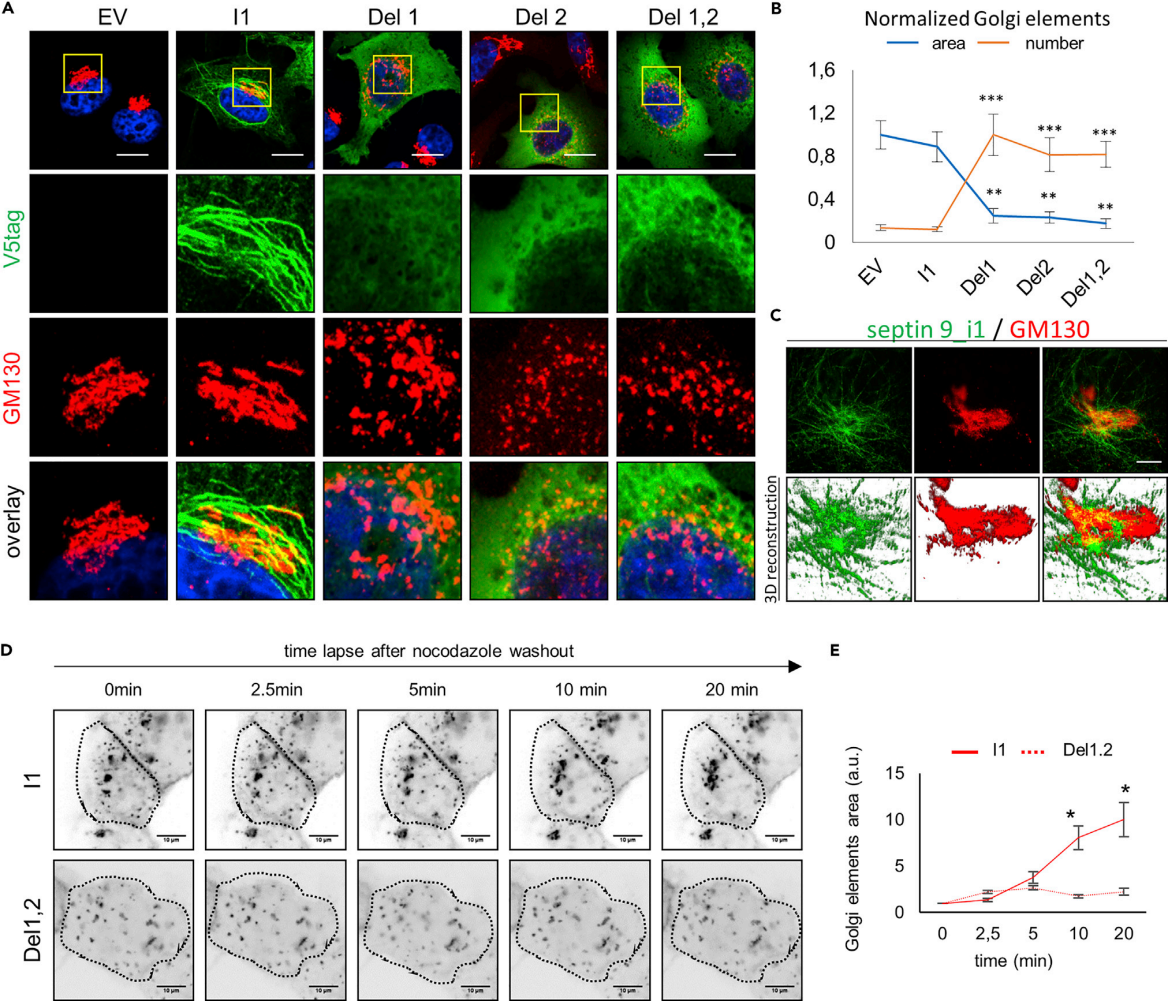
Septin 9 Localizes to Golgi and Is Required to Ensure Its Compact Morphology (A) HeLa cells transfected with an empty vector (EV), septin 9_i1 (I1), septin 9_del1 (Del1), septin 9_del2 (Del2), or septin 9_del1,2 (Del1,2) for 48 h and then fixed and stained for Golgi with GM130 (red) and V5tag (green). Squares indicate the area shown at higher magnification below. Scale bar, 10 μm. (B) Line graph representing the normalized number and size of Golgi elements. Values are mean ± SEM from 15 cells under each condition for three independent experiments. **p < 0.001, ***p < 0.0001 (Student's t test). (C) Stimulated emission depletion microscopy (STED) high-resolution microscopic images of HeLa cells transfected with septin9_i1 (I1) and then fixed and stained for GM130 (red) and V5tag (green). Images are shown in 3D reconstructions below. Scale bar, 5 μm. (D) MDCK cells stably expressing septin 9_i1 or septin 9_del1,2, transfected with KDE-GFP for 24 h, and subjected to nocodazole washout. Images represent video frames illustrating the reassembly of Golgi after the removal of nocodazole. (E) Dotted lines indicate the cell periphery. Line graphs to the right represent fold increases in Golgi elements relative to time 0 during three experiments. Scale bar, 10 μm. *p < 0.05 (Student's t test).

A significant increase in EE and ER markers was observed in the perinuclear region of septin 9_i1-expressing cells when compared with empty vector (EV) and septin 9 mutants, which were similar (Figures S5A–S5E). The most striking observation of the deletion of PB domains was on the Golgi structure (Figures 5A and 5B). In cells expressing septin 9_i1, we found the normal phenotype of a compact Golgi embedded in higher-order structures of septin 9_i1 filaments (Figures 5A and 5C), which contained septins 2, 6, and 7 (Figure S5F). Strikingly, the deletion or mutation of any of the PB domains led to Golgi fragmentation (Figures 5A, 5B, and S5G).

Assembly of the Golgi apparatus is dependent on microtubule polymerization (Miller et al., 2009). The depolymerization of microtubules, typically under nocodazole treatment, results in Golgi fragmentation; removal of the nocodazole enables the re-polymerization of microtubules and Golgi reassembly. We thus took MDCK cells stably expressing septin 9_i1 and septin 9_del1,2 and treated them with nocodazole to induce Golgi fragmentation. The nocodazole was then washed out and Golgi reassembly monitored (Figure 5D). In septin 9_i1 cells, the scattered Golgi elements reassembled normally within 20 min (Figures 5D and 5E), but they did not in septin 9_del1,2 cells (Figures 5D and 5E), even after much longer periods of time (Figure S5H).

The aforementioned observations suggest a function for septin filaments in Golgi assembly that is dependent on septin 9 PB domains.

### Mutations of PB Domains Cause Golgi Fragmentation, but Not Septin 9 Dissociation from the Membrane

**Figure 6:**
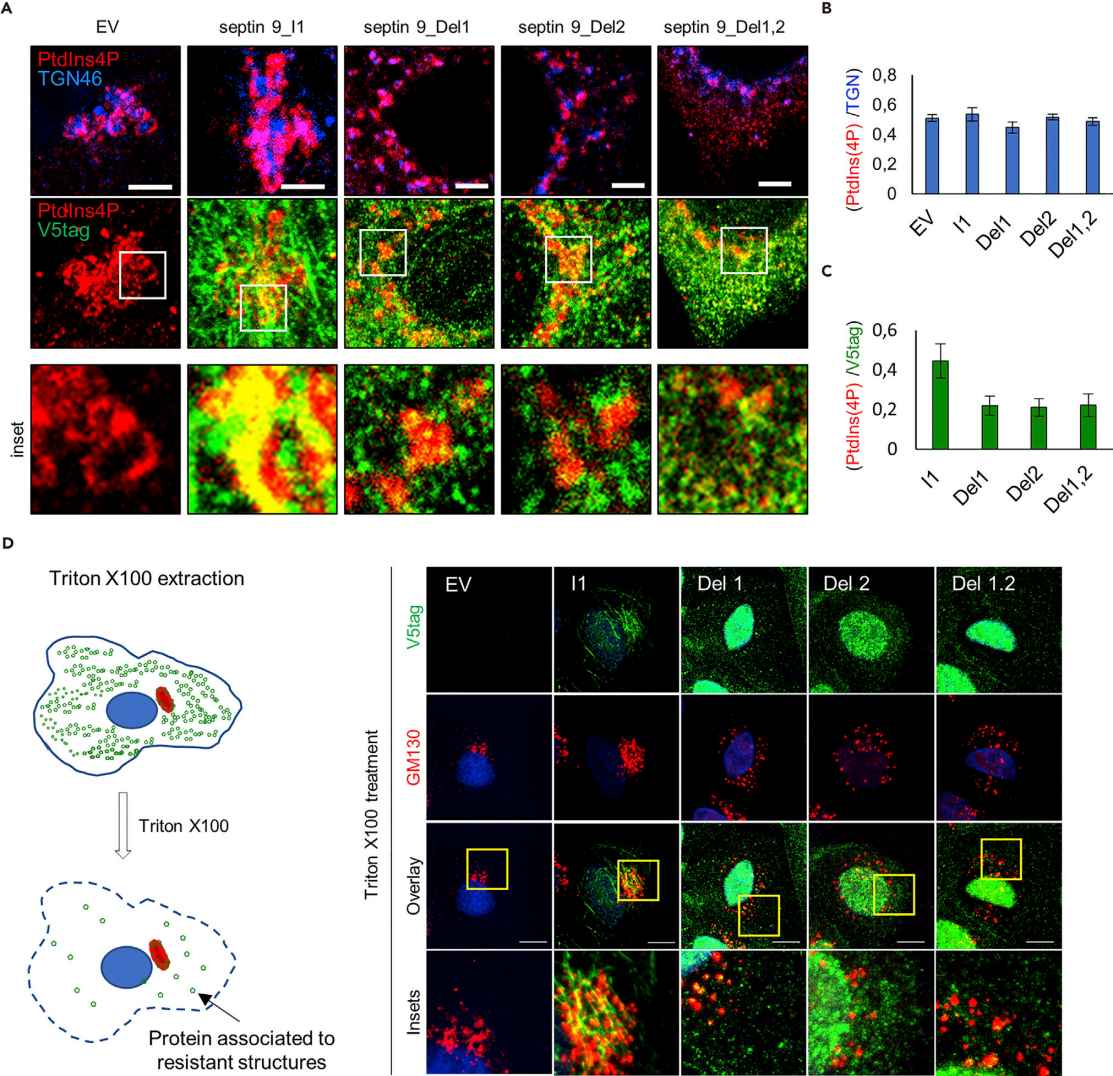
PBs Are Required for the Specific Recruitment of Septin 9_i1 to the Golgi (A) HeLa cells were transfected with an empty vector (EV), septin 9_i1 (I1), septin 9_del1 (Del1), septin 9_del2 (Del2), or septin 9_del1,2 (Del1,2) for 48 h before being fixed and stained for PtdIns(4)P (red), TGN46 (blue), and V5tag (green). Squares indicate the area shown below at higher magnification. Scale bar, 3 μm. (B) Bar graph represents Pearson's correlation coefficient (Rr) analysis of PtdIns(4)P and TGN46. Values are mean ± SEM of 10 cells under each condition from three experiments. (C) Bar graph representing Pearson's correlation coefficient (Rr) analysis of PtdIns(4)P and V5 tag. Values are mean ± SEM of 10 cells under each condition from three experiments. (D) Left, schematic representation illustrating cell extraction with Triton X-100. On the right, MDCK stably transfected with EV, septin 9_i1 (I1), septin_9 del1 (Del1), septin_9 del2 (Del2), or septin_9 del1.2 (Del1,2) were grown for 24 h on coverslips. The cells were then extracted using cold PBS buffer containing Triton X-100 at 0.1% for 30 s and then fixed and stained for V5tag (green) and GM130 (red). Scale bar, 10 μm.

Our data suggested that a lack of PB domains might cause a loss of the specific binding of septin 9 to PtdIns(4)P (Figure 2). We decided to monitor the interaction between septin 9 and PtdIns(4)P within cells. HeLa cells were therefore transfected with the cDNAs of EV, septin 9_i1, and Del1, Del2, or Del1,2 and stained for V5 tag, TGN46 (Golgi marker), and PtdIns(4)P (Figures 6A and S6A).

PtdIns(4)P was strongly co-localized with compact Golgi in EV and septin 9_i1, as had been expected (Dippold et al., 2009). In cells transfected with the Del1, Del2, or Del1,2 constructs (Figure 6A), the Golgi was fragmented, as seen previously, but no significant difference in co-localization between TGN and PtdIns(4)P was noted (Figure 6B). However, we observed a non-significant decrease in the co-localization of septin 9 with PtdIns(4)P (Figure 6C) that varied between the different transfected constructs. PB-deleted mutants still displayed strong signals on small spherical compartments, which were possibly Golgi ministacks, vesicles, or other structures (Figures 6A and S6A). To determine whether the mutant septin 9 proteins were associated with these membrane structures, we permeabilized the cells and removed the soluble proteins before fixation. We observed that the septin 9 mutated protein signal remained on intracellular structures (Figure 6D). This observation suggests that a lack of PB domains does not prevent the binding of mutant proteins to possible membrane structures, in line with our *in vitro* assays that revealed the ability of the mutants to bind to membranes (Figure 2). To further test this finding, a subcellular fractionation assay was performed (Figure S6B) and showed that septin 9_i1 always peaked with the Golgi marker, whereas septin 9_del1,2 had a more spread out signal, suggesting that it probably bound to other membranes, including fragmented Golgi elements (Figure S6B).

Taken together, our results suggest that PB1 and PB2 restrict septin 9 binding to PI-containing membranes, such as PtdIns(4)P on Golgi, but are not directly responsible for septin membrane association. They play a major function in septin complex assembly and subsequent organelle structuration.

### Endogenous Septin 9 Localizes to Golgi and Regulates Its Compactness and Functionality

**Figure 7:**
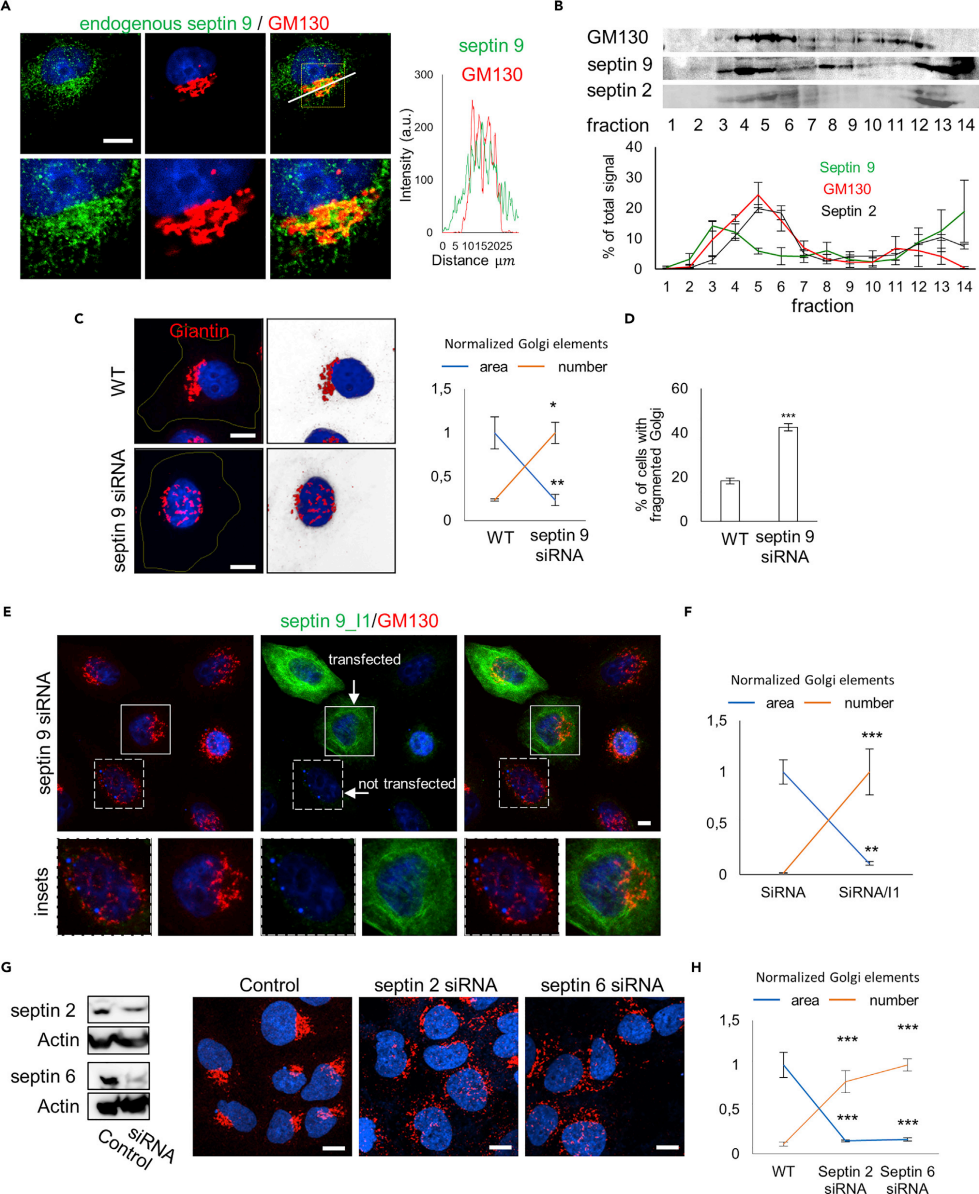
Septin 9 Localizes to Golgi and in the Same Way as Septins Is Required to Ensure Its Compact Morphology (A) Huh7 cells fixed and stained for septin 9 and GM130. Scale bar, 5 μm. On the right, line profile of the white line shown in the merge image. (B) Huh7 cells were grown for 48 h before being subjected to a subcellular fractionation assay and analyzed with western blot for septin 9 and GM130 and septin 2. The line graph below shows densitometry analysis of the subcellular fractionation assay. Values are mean ± SEM from three independent experiments. (C) Septin 9 siRNA and control cells stained for giantin (red). Cells are shown in 2D images with a black background and in 3D reconstruction images with a white background. Scale bar, 10 μm. The right line graph represents the normalized size and number of Golgi elements. Values are mean ± SEM of 150 cells from three independent experiments. *p < 0.05, **p < 0.001 (Student's t test). (D) Percentage of cells with fragmented Golgi obtained from three independent experiments. At least 800 cells were counted for each condition. Values are mean ± SEM. ***p < 0.0001 (Student's t test). (E) Septin 9 siRNA cells were transfected with septin 9_i1 and stained for GM130 (red) V5tag (green). Squares indicate the area shown at higher magnification below. Scale bar, 10 μm. (F) Bar graph representing the size and number of Golgi elements in 20 cells from two independent experiments performed as described in (E). **p < 0.001, ***p < 0.0001 (Student's t test). (G) Huh7.5 cells transfected with septin 2 siRNA or septin 6 siRNA for 48 h were analyzed with western blot (left) and confocal microscopy for GM130 (red) (right). Scale bar, 10 μm. (H) Line graph representing the normalized size and number of Golgi elements in 10 cells from two independent experiments. ***p < 0.0001 (Student's t test).

The transfection of our septin 9 mutant constructs, which had a dominant-negative effect on endogenous septin 9, could be the reason for Golgi fragmentation. We therefore studied this endogenous septin 9 and found that it also co-localized with Golgi (Figure 7A). This observation was likewise supported by a subcellular fractionation assay (Figure 7B) in which both septin 2 and septin 9 peaked with GM130 (Figure 7B). These results suggest that endogenous septin 9 is present in septin structures associated with the Golgi apparatus.

We next worked with a cell line stably transfected with septin 9 small interfering RNA (siRNA) (Figure 7C). In control cells, giantin, which is also a Golgi marker, displayed polarized and compact features, i.e., enriched on one side of the nucleus (Figure 7C). However, in the septin 9 siRNA cells, we observed a fragmentation of the Golgi (Figure 7C). Interestingly, this fragmentation could be rescued by transfection with septin 9_i1 (Figures 7E and 7F), but not with the PB-deleted constructs (Figures S7A and S7B). Here again, we performed the nocodazole washout assay, which confirmed that Golgi reassembly is dependent on endogenous septin 9 (Figure S7C), which therefore plays an important role in maintaining the compact structure of the Golgi.

Our overall data suggest that septin 9 (endogenous and septin 9_i1) is incorporated into filaments that are critical to the Golgi structure. As septin 2 and septin 6 are involved in these filaments, they should also control Golgi compactness. We accordingly found that their depletion by a specific siRNA promoted Golgi fragmentation (Figures 7G, 7H, and S7E). These data reinforced our model in which septin complexes are incorporated into the filament structures that are associated with the Golgi membrane and control its compactness.

Finally, fragmentation of the Golgi apparatus is known to impede secretory pathways (Xiang et al., 2013, Lavieu et al., 2014, Joshi et al., 2015). We thus probed whether septin 9 depletion (Figures S8A and S8B) impeded these pathways. We first used a Venus-tagged neuropeptide Y construct (NPY-Venus) as a model of secreted protein; we determined its level of secretion in the cell culture medium and lysates. We found that septin 9 depletion resulted in a significant decrease of secreted NPY-Venus (Figure S8C). We next studied the trafficking of a temperature-sensitive variant of the vesicular stomatitis virus G protein tagged with GFP (tsVSVG-GFP) (this protein remains in the ER at 40°C but enters the secretory pathway when the temperature is lowered to 32°C; Presley et al., 1997). Control and septin 9-depleted cells were transfected with tsVSVG-GFP and fixed at different time points after the temperature was lowered from 40°C to 32°C (Figure S8D). After 2 h, the protein signal in the Golgi area was greatly reduced in control cells, whereas it remained intact in septin 9-depleted cells, suggesting a defect in intracellular trafficking. Taken together, these data indicate that septin 9 plays a critical role in Golgi compactness and its subsequent function in cellular trafficking.

## Discussion

Septins belong to the family of GTPase proteins that assemble into macrostructure filaments, which are important to the membrane-remodeling processes. Here we have shown that septin 9 is particularly necessary for Golgi structure and function, as the absence of septin 9 provokes Golgi dissociation and impairs secretory pathways. Our results support the idea that septin 9 and other septins (such as septin 2 and septin 6) form a filament matrix that harbors Golgi stacks.

The association of septins with membranes was previously thought to be specifically mediated by an interaction between their polybasic domain PB1 and PI lipids (Pan et al., 2007, Zhang et al., 1999, Tanaka-Takiguchi et al., 2009, Casamayor and Snyder, 2003). Here we found that septin membrane association was much more subtly regulated. First, we identified the presence of a second polybasic domain (PB2) in septin 9, which is conserved among the different human septins and regulates the interaction between septin and PI lipids. Second, we identified AH structures adjacent to PB1 and PB2, which probably mediate the physical association of septin 9 with membranes. Our results support the idea that PB1 and PB2 may act together to restrict the binding of these AHs of septin 9 to PI-containing membranes, and particularly to non-positively curved membranes. This conclusion is in line with the *in vivo* accumulation of septins in specific plasma membrane ingressions or concavities, such as cleavage furrows (Spiliotis and Nelson, 2006). Finally, based on our *in vitro* observations, MD (Figure S2A), and modeling of the interaction between the septin 9 complex and PtdIns(4)P-containing membranes (Figure 2A), PB1 appears to be more closely involved in regulating septin 9 membrane-binding specificity than PB2, but both are critical for septin filament assembly.

Whether septin 9 acts in a cellular context in the monomeric form is not known. Apart from the septin 9 isoform septin 9_i4, which has been found in non-filament structures (Chacko et al., 2005), most septins have been so far been reported as being incorporated in filamentous structures, and whether they exist under monomeric form is also unknown. Despite our *in vitro* studies having been performed with the monomeric septin 9, our results provided information on how septin 9 membrane-binding determinants might influence the localization of septin oligomers to membranes. These results are consistent with a recent report on the presence of AH structures in oligomeric septin filaments, capable of sensing macroscopic curvatures (Cannon et al., 2019).

Based on our results, we propose that septin 9 binds membranes with AHs that can strongly associate to the membrane. In this setting, PB domains may interact with PI lipids to dock the protein on specific organelles, thereby preventing non-specific binding. This mechanism would enrich the protein on the membrane thanks to the PBs, and stabilize it through the action of AHs.

It is not yet clear how septins control the Golgi structure. One hypothesis is that the PB domains contribute to enriching septin 9 or octameric complexes in Golgi elements. Assembly of the octamers would form the septin filaments, which will in the meantime have brought different Golgi elements close enough to promote their fusion, e.g., by SNARE proteins. Under this hypothesis, a septin matrix will form a structure embedding the Golgi elements. This model is consistent with our experiments on Golgi reassembly after nocodazole treatment: this reassembly occurred with septin 9_i1, whereas with Del1,2 (Figures 5D, 5E, S5H) or septin 9 depletion (Figure S7C) it did not. Consistent also with our model, the Golgi is fragmented by the depletion of septin 2 or 6. Hence our study has revealed the importance of higher-order septin structures in Golgi homeostasis. As microtubules and actin filaments are also known to maintain Golgi structure and interact with septins (Miller et al., 2009, Egea et al., 2006, Kondylis et al., 2007, Fung et al., 2014), it is plausible that septins are engaged in hybrid filaments with microtubules and actin to maintain Golgi structure.

On other organelles, we observed that PB1 and PB2 affected ER and EE organization, which was reminiscent of the role that we described for septin 9 in lipid droplet dynamics; interestingly, septin filaments have been seen to be prominent around large lipid droplets (Akil et al., 2016). This particular localization of septin higher-order structures to sites of micron-scale membrane curvature is emerging as an important feature of organelle dynamics (Cannon et al., 2019).

Finally, septin structures have been proposed to act as a physical barrier against the non-specific docking of vesicles on the active zone of the synapse (Yang et al., 2010). Thus septin structures may also behave as physical barriers that prevent the collapse of Golgi stacks or their connection to other organelles. Our findings on the role of septin 9 in the Golgi apparatus could probably be extended to other organelles and offer a paradigm for the structural and biological functions of septins.

### Limitation of the Study

One limitation of this study is that the septin 9 protein studied *in vitro* is in monomeric form, whereas it is unknown whether it can exist in such form in cells.

## Methods

All methods can be found in the accompanying Transparent Methods supplemental file.

## Supplemental Information

Document S1. Transparent Methods, Figures S1–S8, and Table S1
