# Septin 9 has Two Polybasic Domains Critical to Septin Filament Assembly and Golgi Integrity

**DOI:** 10.1016/j.isci.2020.101042

**Published:** 2020-04-18

**Authors:** Mohyeddine Omrane, Amanda Souza Camara, Cyntia Taveneau, Nassima Benzoubir, Thibault Tubiana, Jinchao Yu, Raphaël Guérois, Didier Samuel, Bruno Goud, Christian Poüs, Stéphane Bressanelli, Richard Charles Garratt, Abdou Rachid Thiam, Ama Gassama-Diagne

## Main Text

(iScience *13*, 138–153; March 29, 2019)

Due to errors in figure preparation, the inset panels for Figure 6A did not match the main images and the Septin9_Q1 panel in Figure S5G did not correspond to the merged image. The figures have now been corrected.

Furthermore, the figure legend in figure 2C was incomplete. The following sentence has been added:

“Figure 2C depicts representative data from a total of two individual replicates. Quantification of the data is challenging to perform due to the fact that septin 9 in isolation is prone to spontaneous cleavage. Additional bands seen in (C) likely represent protein cleavage products." The legend for figure 2D has been corrected as follows: “(D) Bar graph representing the percentage of protein in the top fraction (bound protein) from an analysis of the blots presented in (C).”

The authors apologize for any inconvenience.

**Figure:**
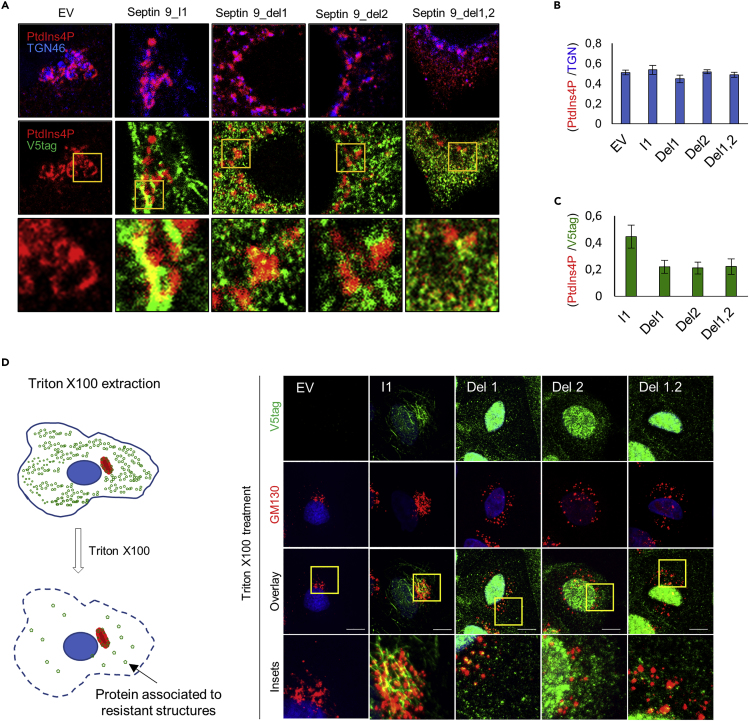
Figure 6. PBs Are Required for the Specific Recruitment of Septin 9_i1 to the Golgi (corrected)

**Figure:**
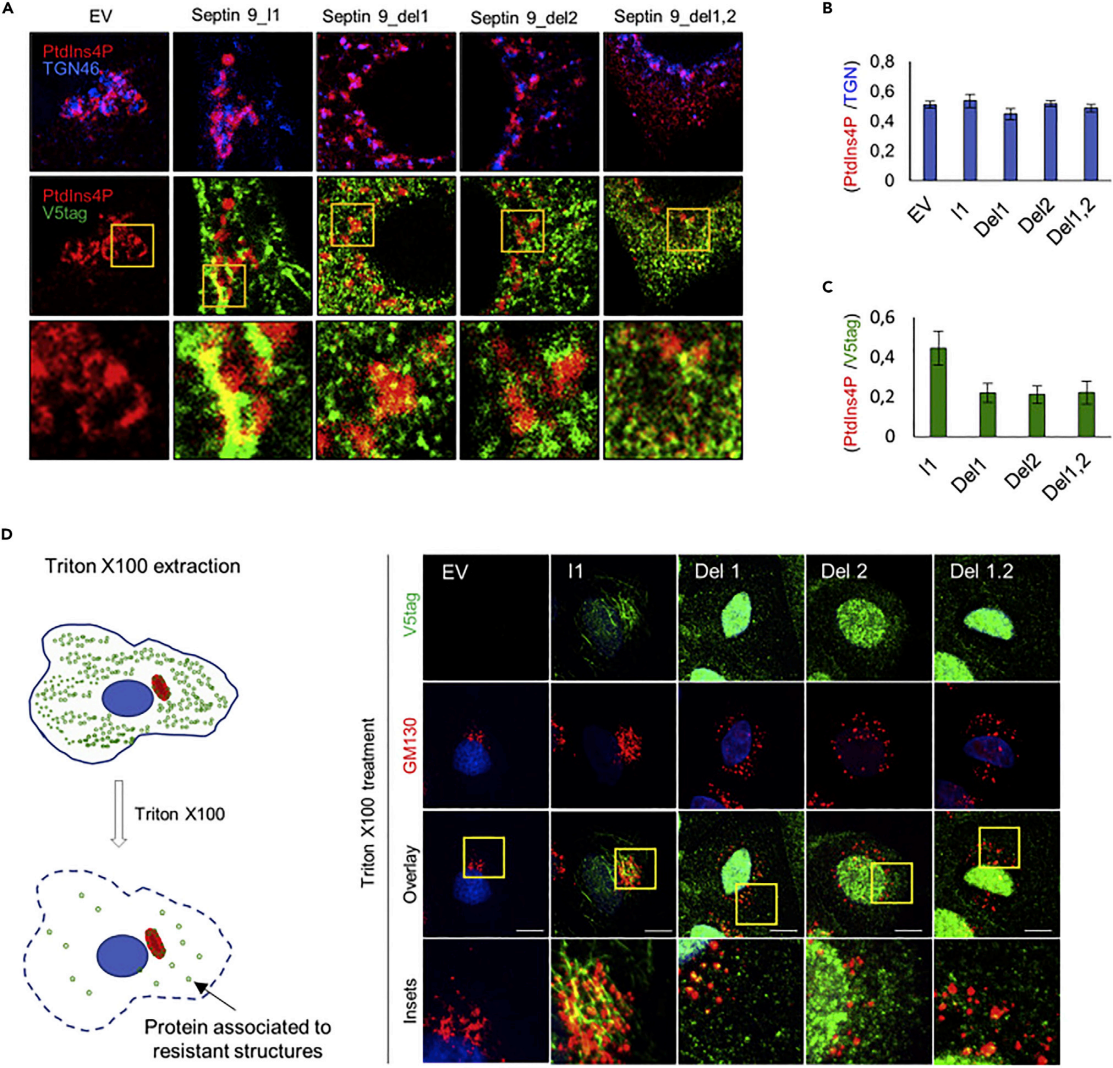
Figure 6. PBs Are Required for the Specific Recruitment of Septin 9_i1 to the Golgi (original)

**Figure:**
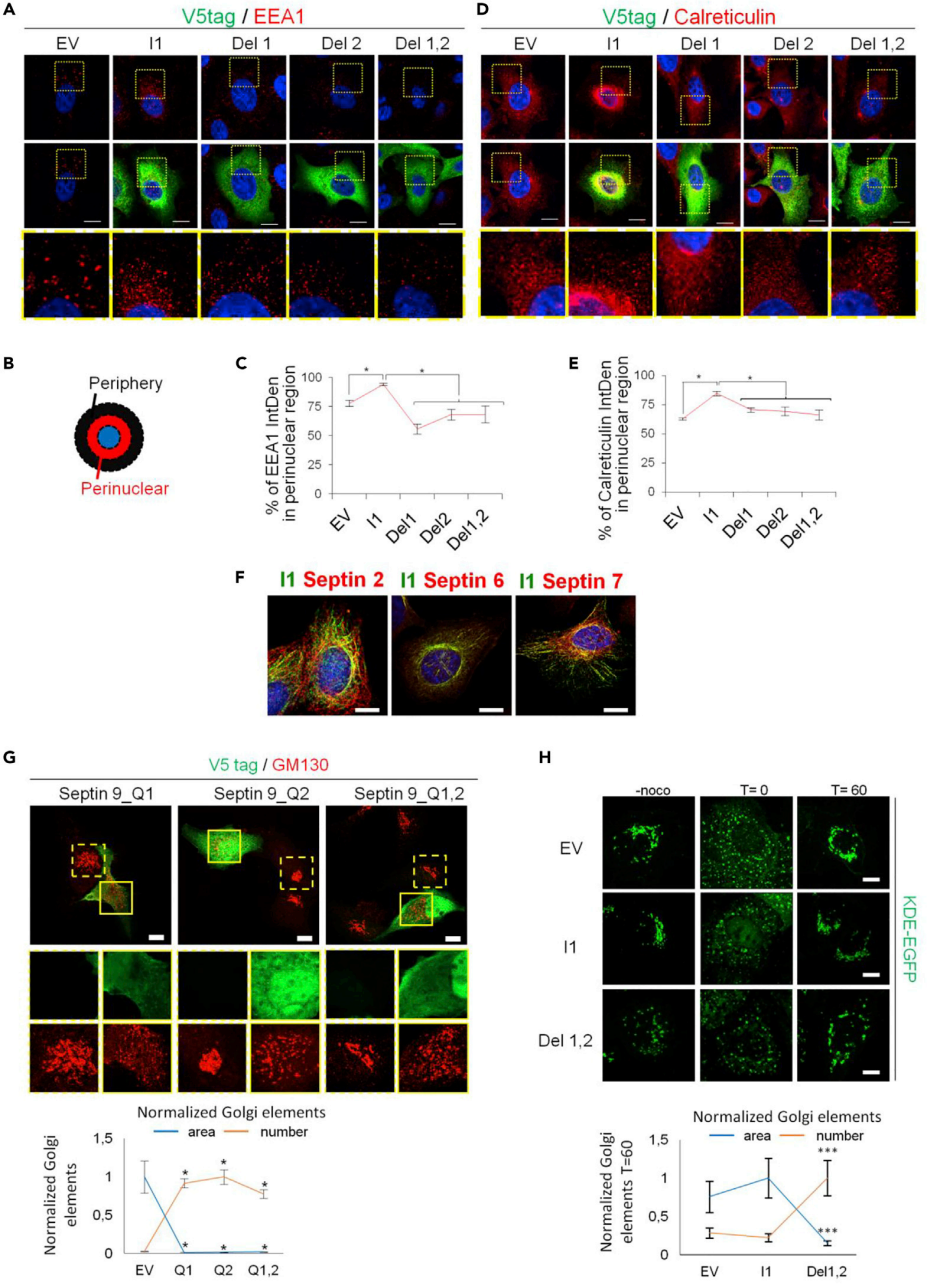
Figure S5. Mutated septin 9 i1 are incapable of having the effect of septin 9_i1 on Golgi, ER and EE compartments, Related to Figure 5 (corrected)

**Figure:**
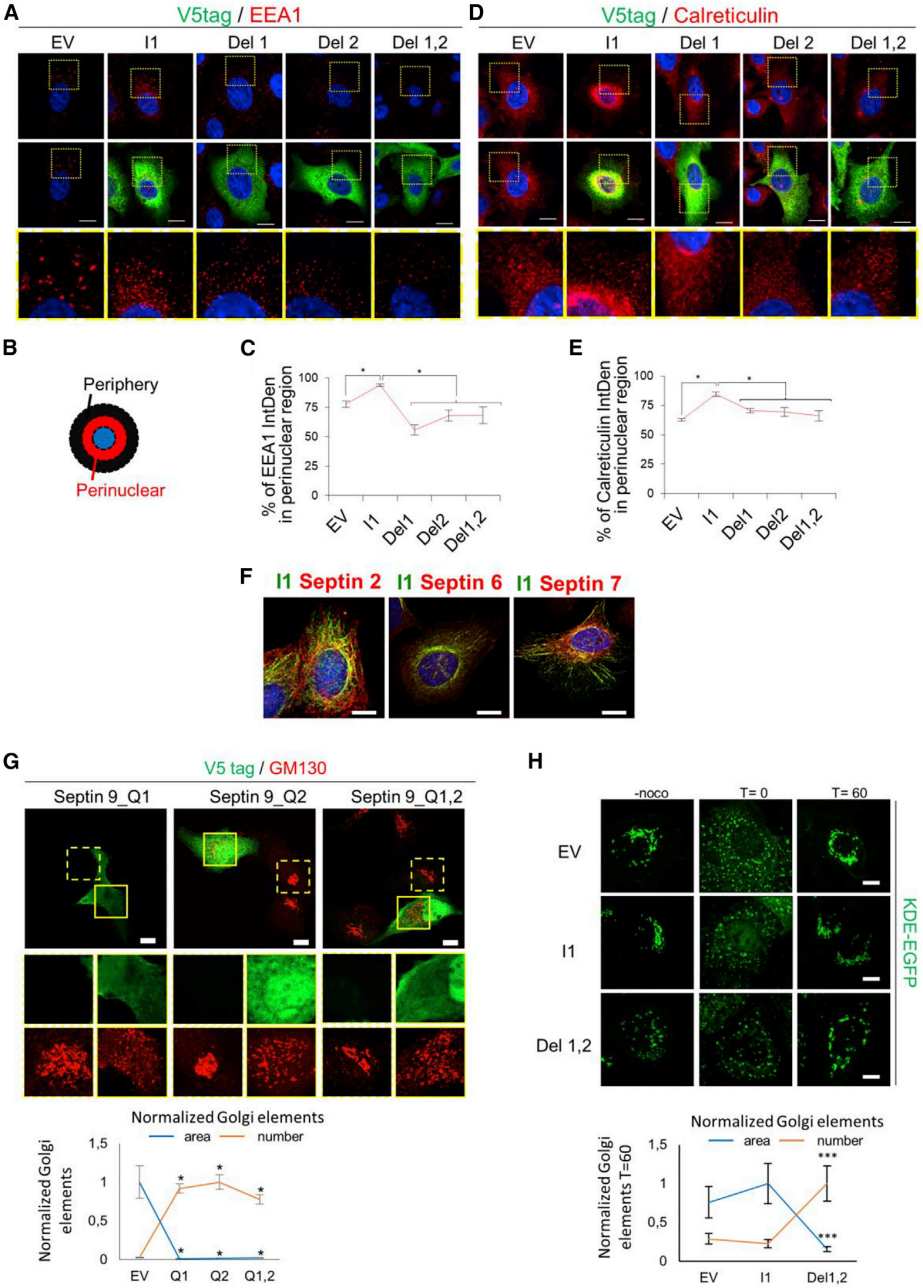
Figure S5. Mutated septin 9 i1 are incapable of having the effect of septin 9_i1 on Golgi, ER and EE compartments, Related to Figure 5 (original)

